# The relationship between parenting styles and self-esteem of medical students with age and gender as moderators

**DOI:** 10.1097/MS9.0000000000002432

**Published:** 2024-08-06

**Authors:** Fahad Gul, Khawar Abbas, Sajeel Saeed, Tehseen Haider, Sardar Noman Qayyum, Samim Noori

**Affiliations:** a Department of Psychiatry, Rawalpindi Medical University; b Department of Medicine, Rawalpindi Medical University, Rawalpindi; c Department of Psychiatry, Bacha Khan Medical College, Mardan, Pakistan; d Nangarhar University, Faculty of Medicine, Nangarhar, Afghanistan

**Keywords:** authoritarian, authoritative, parenting styles, permissive, self-esteem

## Abstract

**Introduction:**

Among various factors that determine an individual’s self-esteem, parenting styles play a significant role. This study investigates the link between parenting styles and self-esteem among medical students while exploring the role of age and gender in this context.

**Methodology:**

A cross-sectional study was carried out among medical students from December 2020 to March 2021. An online survey was prepared using the Rosenberg Self-Esteem Scale and Parental Authority Questionnaire-Short Version, and students were asked to fill it out. Data were gathered from 255 students by simple random sampling technique, of which 230 forms were filled. SPSS version 26.0 was used to enter and analyze the data. One sample *t*-test, Pearson Correlation, and Hierarchal regression analysis were applied. AMOS version 26.00 was used for confirmatory factor analysis.

**Results:**

Out of 230 participants, 60% of the sample population experienced an authoritative parenting style. Authoritarian and authoritative parenting styles were significantly correlated with self-esteem. Females who experienced authoritative parenting and males who experienced authoritarian and permissive parenting styles had higher self-esteem than their respective counterparts.

**Conclusion:**

Authoritative parenting was the most common and the only parenting style with a statistically significant positive correlation with self-esteem. This study further highlights the importance of consistent parental supervision and open communication in determining an individual’s self-esteem.

## Introduction

Highlights

- The relationship between various parenting styles and the self-esteem of Pakistani medical students was explored.
- The authoritative parenting was the most common and it positively correlated with self-esteem. Females had higher self-esteem under authoritative parenting as compared to males.
- The authoritarian parenting negatively correlated with the self-esteem of both male and female medical students.
- Permissive style and age positively correlated with self-esteem of female medical students while negatively with self-esteem of male medical students.
- The study recommends that authoritative approach to foster positive self-esteem in medical students.

The contemporary challenge in child development research lies in comprehending the profound impact of parenting styles on children’s self-esteem. Currently, there is a critical issue concerning the understanding and implementation of effective parenting styles that positively influence children’s self-esteem. Self-esteem refers to a person’s judgment towards himself, a person’s perception of himself both physically and psychologically^1^. Parenting styles relate to a collection of actions and patterns that parents use while parenting their children. Additionally, it encompasses spoken and nonverbal communication between parents and children in various contexts. Different parenting styles, such as positive parenting and affectionless parenting, have been discussed over the years. However, the Baumrind typology has gained matchless popularity across the globe^2^.

Baumrind discusses two dimensions to capture parenting styles; demandingness and responsiveness^3^. Strictness or demandingness refers to parental control or power over their children. Warmth or responsiveness refers to affection, care, and communication between parents and children. Concerning these two dimensions, Baumrind established a conceptual framework for three distinct parenting styles (authoritative, authoritarian, and permissive). Later, Maccoby and Martin reconceptualized this typology by admixing parental responsiveness and control to introduce ‘neglectful parenting’^3^.

Authoritarian parents show dictatorial, nonwarmth, and strict behavior towards their children and demand complete obedience from them^4^. They create an environment limited to cold and unidirectional communication, and expect their kid to adhere to their stringent disciplinary guidelines at all costs. In other words, they are nonresponsive and have high demands^4^. Authoritative parents have high needs but are highly responsive at the same time. They have clear, consistent boundaries and have open communication with their children^5^. Permissive parents show warmth and nonstrict behavior; they do not try to change the child’s behavior even when their actions are undesirable. They rarely demand something from their child, do not have any disciplinary rules. In other words, they are highly responsive and nondemanding^5^. Other parenting styles, which include indulgent (low demanding, highly responsive, warmth but not strict) and neglectful (low demanding, low responsive, and neither warmth nor strict), are under study nowadays^6^.

Parental care, support, and affection raise the child with psychological maturity and high self-esteem, whereas over-controlling, overprotection, and strict directions raise the child with emotional deficiency and low self-esteem^7^. Numerous studies have been conducted to determine the effect of parenting styles on a child’s growth and development, and the results indicate that children raised with authoritative parenting are more socially, emotionally, and academically competent than children raised with other parenting styles^8^. Following various types of research conducted in preceding years, authoritative parenting proved to be the most practiced parenting style in most of the Western countries and has shown a significant positive correlation with self-esteem^9^. Contrarily authoritarian parenting negatively correlates with self-esteem^9^. In a study conducted in Iran, children who experienced authoritative parenting have a better self-concept, self-esteem, psychological health, and quality of life than others^10^.

In the last decade, various studies have been conducted in Pakistan on how parenting styles affect a child’s psychological development^11^. However, only some targeted the correlation of measures of parenting styles with self-esteem. According to a study conducted at the Mayo Clinic’s Department of Child and Family psychiatry, self-esteem is adversely correlated with apathetic, abusive, and overly controlling parenting methods^11^. An analysis conducted at the University of Sargodha with a sample of only 100 participants showed a negative correlation between social anxiety and permissive parenting style with self-esteem. Still, no significant correlation was found between authoritative and authoritarian parenting styles^12^.

Knowing all the facts, it is still premature to conclude that the most optimum parenting style is authoritative given the strong influence of culture, environment, and traditions. In an environment where disobeying parents harms the child, such as in African American communities, authoritarian parenting may be as valuable as other parenting styles^13^. An analysis conducted in China suggested authoritative parenting might not be helpful in Chinese families compared to their traditional parenting, which instills the need to work hard and follow strict disciplinary rules^14^.

A meta-analysis of longitudinal research revealed that self-esteem grows throughout infancy, stays stable during adolescence, increases gradually during adulthood, reaches a peak between the ages of 60 and 70, and then drops. This pattern follows irrespective of gender, country, ethnicity, and sample type^15^. On the contrary, Heaven *et al*. observed a decline in self-esteem with time, pointedly in girls. Grade 7 girls showed higher self-esteem than grade 7 boys, but grade 10 girls showed lower self-esteem than grade 10 boys^16^. According to cross-cultural research, males have stronger self-esteem than females, and it grows dramatically with age^17^.

Braza *et al*.^18^ illuminated gender as a moderator in the correlation between parenting styles and child behavior. Quatman and Watson^19^ inferred that boys have more self-esteem than girls in six out of eight domains of self-esteem, while the remaining two do not exhibit any gender difference. Keshavarz and Baharudin^20^ conducted a study on Malaysian children, which manifested a significant positive correlation between both genders’ paternal authoritative parenting style and self-esteem. Still, boys showed a stronger correlation than girls. Gender difference in self-esteem was also demonstrated in adolescents studying in private secondary schools in Karachi, where girls established higher self-esteem than boys^21^. On the contrary, boys showed higher self-esteem than girls in district Mianwali and district Bahawalnagar schools^22^.

Many types of research have been conducted in the West on the relationship between parenting styles and the self-esteem of children, students, and adolescents, but little is known in Asian countries^23^. This study will target the correlation between globally accepted parenting styles and self-esteem in Pakistani culture. In a nutshell, none of the studies in the last decade specifically targeted the correlation between globally accepted parenting styles and self-esteem in Pakistani culture. Additionally, precedent studies poorly illuminate the influence of gender and age as moderator variables in parenting styles and self-esteem in Pakistani culture. Therefore, this research aims to raise awareness of the optimum parenting style for the self-esteem of a child in Pakistani culture by providing a statistically backed analysis of the most common parenting style among medical students and its impact on their self-esteem while studying the effect of gender and age as moderator variables.

## Methodology

### Study design and data collection

From 1 December 2020 to 31 March 2021, a cross-sectional study was undertaken among medical students aged 17–25. The data was gathered irrespective of gender. The study’s inclusion criteria was: a) undergraduate medical students willing to participate. The study’s exclusion criteria was: a) undergraduate students whose one or both parents died before their birth, b) students who were adopted, and c) students not willing to participate.

Using the WHO sample size calculator, the calculated sample size was 255 with a population size of 1200, 5% margin of error, 95% CI, and an expected population proportion having high self-esteem of 0.7. The formula is given as


$$n=\frac{z_{\frac{1-\alpha }{2}}^{2}P(1-P)N}{d^{2}(N-1)+z_{\frac{1-\alpha }{2}}^{2}}$$

The participants were chosen from the study population using nonrandom convenient sampling. For data gathering, a questionnaire was developed using Google Forms. It had three components: demographic details, Rosenberg Self-Esteem Scale (RSE), and Parental Authority Questionnaire-Short Version. That questionnaire was sent to 255 participants via E-mail, Facebook, and WhatsApp. Of 255, 230 forms were filled, giving us a response rate of 90%.

### Ethical approval

Ethical approval was obtained (PSY-19-45-21) from the Institutional Research Forum. Consent was obtained from each participant individually. The data collection procedure was carried out in compliance with institutional and national ethical guidelines. Furthermore, permission was granted by Hussain Alkharusi on our request to use his Parental Authority Questionnaire-Short Version (PAQ-S)^24^ in our study. For Rosenberg’s Self-Esteem Scale^25^, permission was granted by Rosenberg’s Family. The manuscript followed STROCCS guidelines for reporting a cross-sectional study^26^.

### Measures

Demographic details, including gender, age, year of study, and socioeconomic status of the family, were obtained from each participant. This study used two scales: the Rosenberg Self-Esteem Scale (RSE) and Parental Authority Questionnaire-Short Version (PAQ-S).

#### Parental authority questionnaire-short version (PAQ-S)

Alkharusi *et al*.^24^ created the Parenting Authority Questionnaire-Short Version (PAQ-S) to assess authoritative, authoritarian, and permissive parental styles or discipline methods from the viewpoint of a kid of any age.

This scale is a self-report measure based on Baumrind’s Parenting Styles Model^27^ and a short version of the Parental Authority Questionnaire developed by Buri^28^. The concurrent validity of this scale with the extended version PAQ was highly significant (*P*<0.001), and internal consistency, as well as reliability scores of this scale, were comparable to scores of extended version PAQ^24^.

This poll employed a five-point Likert scale, with strongly disagree=1, severely disagree=2, neutral=3, agree=4, and strongly agree=5 for each of the 20 items. The scale includes seven questions indicating authoritative and authoritarian parenting styles and six items indicating permissive parenting styles. No item requires reverse scoring. The mean score for each subscale was used to determine the participant’s prevailing parenting style.

#### Rosenberg self-esteem scale (RSE)

Rosenberg developed Rosenberg’s Self-Esteem Scale (RSE) in 1965, a self-report instrument that measures self-esteem by assessing good and bad feelings about oneself^25^. It is a 10-item scale with a four-point Likert scale, with ‘Strongly Disagree’ receiving one point, ‘Disagree’ receiving two points, ‘Agree’ receiving three points, and ‘Strongly Agree’ receiving four points. Reverse scoring is applied to items 2, 5, 6, 8, and 9. Add up the scores for each of the 10 things.

### Data entry and analysis

Data were entered and analyzed using SPSS version 26.0. McDonald’s omega coefficient (α), and Cronbach’s alpha coefficient (α) were computed to test for the internal consistency of both the questionnaires. The factorial validity of the Parental Authority Questionnaire-Short Version (PAQ-S) and Rosenberg Self-esteem questionnaire in the existing population was investigated by exploratory factor analysis using the principal axis factoring method. Then, using AMOS version 26.00, confirmatory factor analysis was performed. The following criteria were used to support the fit of the Parental Authority Questionnaire and Rosenberg Self-Esteem Scale: standardized root mean square residual (SRMR <0.08); comparative fit index (CFI> 0.90); root mean square approximation error (RMSEA<0.08). Each attribute was quantified using descriptive statistics such as percentage, frequency, mean, and SD. Pearson correlation was utilized independently for each gender to determine the association between various parenting techniques and self-esteem.

Hierarchal multiple regression analysis was used to check the association between parenting styles and self-esteem, controlling age and gender. The interactional effect of authoritative style × age, authoritative style × gender, authoritarian style × age, authoritarian style × gender, permissive style × age, and permissive style × gender with self-esteem were also tested. The predictor variables were entered in three steps: (1) Age, Gender; (2) Authoritative style, Authoritarian style, Permissive style; (3) six interactional terms authoritative style × age, authoritative style × gender, authoritarian style × age, authoritarian style × gender, permissive style × age, and permissive style × gender.

## Results

A total of 230 filled questionnaires were analyzed. Of these, 84 (36.5%) were males, and 146 (63.5%) were females. 64 (28.3%) from 1st year, 38 (16.5%) from 2nd year, 86 (37.4%) from 3rd year, 26 (11.3%) from 4th year, and 149 (6.5%) individuals from the final year participated in our research. Forty-nine (21.3%) individuals were in the age group of 17–19, 148 (64.3%) in 20–22, whereas the remaining 33 (14.3%) participants were in the age group of 23–25.

### Correlations and disparities in gender distributions for the variables included in the study

One hundred thirty-eight (60%) participants experienced an authoritative parenting style from their parents, 67 (29%) experienced an authoritarian, whereas the remaining 25 (11%) participants experienced a permissive parenting style. Table 1 shows the mean, SD, and one-sample *t*-test of the model variables.

**Table 1:** Means, SD, and the one-sample *t*-test of the model’s variables

Variables	M (95% CI)	SD	*t*
Authoritative style (%)	72.06 (70.06–74.06)	15.38	71.06**
Authoritarian style (%)	61.48 (59.33–63.62)	16.51	56.47**
Permissive style (%)	53.48 (51.69–55.26)	13.74	59.02**
Male age	21.19 (20.86–21.51)	1.50	128.72**
Female age	20.75 (20.49–21.01)	1.62	154.67**
Self esteem	28.77 (28.21–29.34)	4.39	99.39**

Note: *Significance in one sample *t*-test with a *P*-value less than 0.05.

**Significance in one sample *t*-test with a *P*-value less than 0.001.

Participants’ means for authoritative parenting styles, self-esteem, and female age were below the midpoint, whereas permissive parenting styles, authoritarian parenting styles, and male age were above the midpoint. One sample *t*-value indicates that the individuals in our study had higher self-esteem, and most of them had found their parents either more authoritative or permissive but less authoritarian.

Table 2 shows the sample variables’ Cronbach’s alpha and gender-wise zero-order Pearson correlations. The authoritative parenting style consisted of seven items (α=0.858), the authoritarian parenting style consisted of seven items (α=0.835), the permissive parenting style consisted of 6 items (α=0.705) whereas self-esteem consisted of 10 items (α=0.842). McDonald’s omega coefficient of the authoritative parenting subscale was 0.863, the authoritarian parenting subscale was 0.841, the permissive parenting subscale was 0.706 and Rosenberg’s self-esteem scale was 0.851. Authoritative (r=0.283, *P*<0.001) and authoritarian (r=−0.227, *P*<0.001) parenting styles show a positive and negative correlation with self-esteem, respectively. Permissive parenting style (r=−0.038, *P*>0.05) was not significantly correlated with self-esteem by the Pearson correlational model. A positive correlation was found between authoritative style and self-esteem, whereas a negative correlation was found between authoritarian style and self-esteem for both boys and girls. Permissive style and age positively correlated with self-esteem among girls, while a negative correlation was found among boys.

**Table 2:** Bivariate correlations and cronbach’s alpha coefficients of predictor variables and covariates

Variable	Authoritative	Authoritarian	Permissive	Age	Self esteem
Authoritative	1 (0.858)	−0.339**	0.228**	0.037	0.321**
Authoritarian	−0.134	1 (0.835)	−0.338**	−0.058	−0.253**
Permissive	0.068	−0.191	1 (0.705)	−0.075	0.014
Age	−0.116	0.039	−0.004	1	0.009
Self Esteem	0.189	−0.183	−0.143	−0.029	1 (0.842)

Note: Correlation for girls is above the diagonal, and correlations for boys are below the diagonal.

*Significance in Pearson correlation with a *P*-value less than 0.05.

**Significance in Pearson correlation with a *P*-value less than 0.001.

In the parentheses are Cronbach’s alpha coefficients.

The results of the exploratory factor analysis showed that three factors should be retained for the Parental Authority Questionnaire. The result for Bartlett’s test, *χ* ^2^ (190)=1691.77, *P*<0.001, and Kaiser-Meyer-Olkin Measure of sampling adequacy (0.85), also supported the sufficiency and factorability of the data. Using the Varimax method, all the items were scored between 0.38 and 0.81 (Table 3). Confirmatory factor analysis showed satisfactory results for the parental authority questionnaire [*χ* ^2^=402.158 (df=167), SRMR=0.098, RMSEA=0.078] except for the CFI, which was 0.849. Confirmatory factor analysis results for the Rosenberg Self-Esteem scale were also satisfactory [*χ* ^2^=75.948 (df=34), CFI=0.942, SRMR=0.059, RMSEA=0.073].

**Table 3:** Rotated factor loadings of the short versioned parental authority questionnaire

Item	Authoritative	Authoritarian	Permissive
1. ‘Once family policy had been established, my parents discussed the reasoning behind the policy with the children.’	0.51		
2. ‘My parents directed the activities and decisions of the children through reasoning and discipline.’	0.62		
3. ‘As the children in my family were growing up, my parents consistently gave us direction and guidance in rational and objective ways.’	0.72		
4. ‘My parents had clear standards of behavior for the children in our home, but they were willing to adjust those standards to the needs of each of the individual children in the family.’	0.67		
5. ‘My parents gave me direction for my behavior and activities as I was growing up and they expected me to follow their direction, but they were always willing to listen to my concerns and to discuss that direction with me.’	0.81		
6. ‘As I was growing up, my parents gave me clear direction for my behaviors and activities, but they were also understanding when I disagreed with them.’	0.77		
7. ‘As I was growing up, if my parents made a decision in the family that hurt me, they were willing to discuss that decision with me and to admit it if they had made a mistake.’	0.65		
8. ‘Even if their children didn’t agree with them, my parents felt that it was for our own good if we were forced to conform to what they thought was right.’		0.38	
9. ‘Whenever my parents told me to do something as I was growing up, they expected me to do it immediately without asking any questions.’		0.49	
10. ‘My parents have always felt that more force should be used by parents in order to get their children to behave the way they are supposed to.’		0.76	
11. ‘My parents felt that wise parents should teach their children early who is the boss in the family.’		0.69	
12. ‘As I was growing up, my parents would get very upset if I tried to disagree with them.’		0.59	
13. ‘As I was growing up, my parents let me know what behavior they expected of me, and if I did not meet those expectations, they punished me.’		0.62	
14. ‘My parents have always felt that most problems in society would be solved if parents strictly and forcibly dealt with their children when they do not do what they are supposed to.’		0.75	
15. ‘My parents have always felt that children need to be free to make up their own minds and to do what they want to do, even if this does not agree with what their parents might want.’			0.48
16. ‘As I was growing up, my parents did not feel that I needed to obey rules and regulations of behavior simply because someone in authority has established them.’			0.53
17. ‘As I was growing up, my parents seldom gave me expectations and guidelines for my behavior.’			0.40
18. ‘My parents feel that most problems in society would be solved if parents did not restrict their children’s activities, decisions, and desires.’			0.57
19. ‘My parents did not view themselves as responsible for directing and guiding my behavior as I was growing up.’			0.59
20. ‘My parents did not direct the behaviors, activities, and desires of the children in the family.’			0.60

The inter-relationship between authoritative, authoritarian, and permissive parental styles and self-esteem was investigated using hierarchical regression analysis while controlling for age and gender. In step 1, age was negatively associated with self-esteem, indicating that people in late adolescence had more self-esteem than people in early adulthood. Gender was also negatively associated with self-esteem, indicating that males had more self-esteem values than their female counterparts. In the second step, the authoritative parenting style showed a significant positive association with self-esteem, whereas authoritarian and permissive parenting styles significantly negatively correlated with self-esteem. This indicated that females who experienced an authoritative parenting style had more self-esteem than males. In contrast, males who experienced authoritarian and permissive parenting styles had more self-esteem than their female counterparts. The covariate variables explained 10% of the variance in self-esteem. In step 3, the interaction effect of authoritative style × age, authoritative style × gender, authoritarian style × age, authoritarian style × gender, permissive style × age, and permissive style × gender was nonsignificant, showing that the strength of association between different parenting styles and self-esteem did not differ significantly between age and gender. In sum, the model explained 8% of the total variance in self-esteem (Table 4).

**Table 4:** Summary of the hierarchical regression analysis for variables predicting self-esteem

	Self Esteem				
	B	SEB	β	F	Adjusted R^2^
Step 1					
Constant	29.23	3.94			
Age	−0.01	0.19	−0.003		
Gender	−0.39	0.61	−0.04	0.21	−0.007
Step 2					
Constant	30.37	4.52			
Age	−0.04	0.17	−0.01		
Gender	−0.32	0.58	−0.04		
Authoritative style	0.07	0.02	0.25**		
Authoritarian style	−0.05	0.02	0.49**		
Permissive style	−0.05	0.02	−0.35*	6.34**	0.104
Step 3					
Constant	15.57	31.98			
Age	0.75	1.50	0.27		
Gender	−2.46	5.09	−0.27		
Authoritative style	0.17	0.26	0.60		
Authoritarian style	0.13	0.26	0.49		
Permissive style	−0.11	0.30	−0.35		
Authoritative style × age	−0.01	0.01	−0.43		
Authoritative style × gender	0.02	0.04	0.15		
Authoritarian style × age	−0.01	0.01	−0.71		
Authoritarian style × gender	−0.01	0.04	−0.04		
Permissive style × age	0.002	0.01	0.16		
Permissive style × gender	0.02	0.04	0.14	2.94**	0.085

Note: Gender; 0=Boy, 1=Girl.

* Significance in hierarchical regression analysis with a *P*-value less than 0.05.

** Significance in hierarchical regression analysis with a *P*-value less than 0.001.

## Discussion

Parenting style is a set of attitudes based upon the communication and emotions of parents towards their children. Our research showed that most participants had experienced authoritative parenting, and the least experienced parenting style was permissive. These findings are in line with earlier research. In our neighborhood, that is, India, adolescents’ most practiced parenting style was authoritative^29^. Authoritative parenting is also common in other Asian countries, such as Iraq^30^. In Hebrew-literate Arab Bedouin families, a traditional Arab sector, something different was expected in the parent-child relationship from Western and Asian cultures. Still, this study also concluded authoritative style is the most common parenting style^31^.

Our research concluded that self-esteem is positively associated with authoritative parenting and adversely associated with authoritarian parenting but has no association with permissive parenting. Various studies explained that authoritative parenting has beneficial effects on constructing higher self-esteem^1,10,29,31,32^. In contrast, Perez-Gramaje *et al*. and Martinez *et al*. found that permissive parenting was an efficient upbringing style regarding self-esteem development in many aspects^4,33^. Dwairy, in his study, described that authoritarianism had no harmful influences on the self-esteem, emotional, and mental development of a child with average intelligence living in an authoritarian society^34^. Dabiri *et al*.^35^ found no significant correlation between parenting styles and self-esteem.

Demographic variables, that is, gender and age of a child, have moderating effects on the parent-child relationship. Boys, as compared to girls, benefit more from parental attention and participation in developing self-esteem and overall well-being. Moreover, parental rejection and neglect seed emotional instability in girls and antisocial behaviors in boys^36,37^. In addition, a person’s self-esteem might change as they become older^38^.

As our study participants’ age group ranged from late adolescence to early adulthood, hierarchical regression analysis showed a decline in self-esteem with increasing age. In contrast, longitudinal research has found that self-esteem declines throughout infancy and adolescence and then modestly rises in early adulthood^31^. At the same time, others found that self-esteem remains stable during adolescence and increases throughout adulthood^15^. Our research depicted contrary results compared to other studies because, for our part, an emerging adult mostly does not take up a liable role while transitioning from adolescence to adulthood, which includes achieving socioeconomic resilience, engaging in relationships, and making parental identity. All these factors contribute to mature personality development, and lacking these factors contributes to lower self-esteem in emerging adults. Because of not being able to gain full independence from their parents in early adulthood and poor academic performance during university, young adults are unable to build their self-worth.

Furthermore, under authoritative parenting, females developed higher self-esteem than males. This was consistent with the remarkable result of Szkody *et al*.^39^, which indicated that authoritativeness led to higher self-esteem in females while assessing the maternal-daughter relationship. The result was the mother-daughter coordination in household activities, resulting in proximity and supervision from the mother. This resulted in girls having higher self-esteem than boys while transitioning from late adolescence to early adulthood. Our research yielded that authoritarian and permissive parenting styles cause males to have higher self-esteem than females. The possibility behind this result is that in the face of a non-Caucasian cultural context, parental authoritarianism favors males toward self-sufficiency and personal stability in difficult life affairs^40^.

Moreover, parental warmth, an essential dimension in permissive parenting, facilitates the internalization of social norms and values in children^33^. In contrast, Japanese research found that authoritative parenting had the same beneficial impacts on girls’ and boys’ self-esteem and mental health^40^. In the Malaysian population, boys developed higher self-esteem than girls in authoritative families^20^. Bibi *et al*.^41^, on the other hand, elaborated that the gender of children did not play any part in determining the parent-child relationship.

The primary reason for most families to observe authoritative parenting is that it helps develop an environment where a child can quickly move toward positive personality development. Authoritativeness favors securing parent-child bonding effectively, opens the door for discussion, develops a sense of management in the child, and makes an individual socially competent. Creativity is the ability to tackle various problems, which is also the result of authoritative parenting^30^.

### Limitations and recommendations

This research makes a critical addition to our society’s understanding of the link between parenting styles and children’s self-esteem since this research demonstrates that an authoritative parenting style positively correlates with self-esteem. Limitations include a modest sample size (*n*=230) and focusing only on university students, which cannot be generalized to Pakistani cultural settings and backgrounds. To establish more accurate results, large-scale and varied samples are needed in future studies. Moreover, this is a cross-sectional study as it determines etiology and outcome. Therefore, the results should be considered only exploratory in the absence of experimental work.

As the short version of the Parental Authority Questionnaire was utilized in this study, additional studies will help test the validity of the results. They will help generalize the findings obtained by using a short version of PAQ. Also, further examination of its items is required because the scale questions carry long sentences^24^.

While using the RSE as a self-esteem measure, it may not be the best choice to assess deep feelings about one’s self-worth. The elements of self-deception and participant preferences while responding may have impacted the findings. Positive and negative forms of item-wording of the scale need to be altered to construct a single-factor, uni-dimensional scale as Rosenberg initially intended^42^. Additional limitations include sole reliance on students’ perspectives for reporting parenting styles, potential nuance loss in the abbreviated PAQ-S, and overlooking crucial factors like socioeconomic status, family structure, and cultural influences as controls or moderators. Furthermore, the limited variance explanation suggests the presence of other influential factors warranting exploration in future research endeavors. Future research should consider all these limitations to strengthen the validity, reliability, and generalizability of findings on the relationship between parenting styles and children’s self-esteem across diverse cultural and demographic contexts.

## Conclusion

The current research investigates optimal parenting methods in Asian culture, highlighting authoritative parenting as significantly correlated with higher self-esteem. We recommend that parents cultivate authoritative parenting in a child’s rearing to flourish them with high self-esteem. Research suggests that parents should have open communication, especially with their daughters, and be responsive and demanding simultaneously, as strict and nonwarmth behavior impinges on females’ self-esteem more than the males in our culture. Future studies could explore the underlying mechanisms of this relationship across diverse Asian societies to enhance parenting practices effectively.

## Ethical approval

Ethical approval was obtained from institutional research forum of Rawalpindi Medical University with reference no. PSY-19-45-21.

## Consent

Written informed consent was obtained from the patient for publication and any accompanying images. A copy of the written consent is available for review by the Editor-in-Chief of this journal on request.

## Source of funding

This research received no specific grant from funding agencies in the public, commercial, or not-for-profit sectors.

## Author contribution

F.G.: conceptualization, methodology, data curation, investigation, validation, writing – original draft, and writing – review and editing; K.A.: conceptualization, methodology, data curation, investigation, validation, and writing – original draft; S.S.: conceptualization, methodology, validation, visualization, formal analysis, writing – original draft, writing – review and editing, and supervision; T.H.: methodology, data curation, and investigation; S.N.Q.: conceptualization, validation, visualization, writing – original draft; S.N.: writing – original draft and writing – review and editing. All authors approved the final manuscript.

## Conflicts of interest disclosure

The authors declare that they have no known competing financial interests or personal relationships that could have appeared to influence the work reported in this paper.

## Research registration unique identifying number (UIN)

1. Name of the registry: not applicable.
2. Unique identifying number or registration ID: not applicable.
3. Hyperlink to your specific registration (must be publicly accessible and will be checked): not applicable.

## Guarantor

Sardar Noman Qayyum, MBBS, Department of Medicine, Bacha Khan Medical College, Mardan. E-mail: dr.sardarnoman@gmail.com; https://orcid.org/0009-0005-9132-7256.

## Data availability statement

The data used to support the findings of this study are available from the corresponding author upon request.

## Provenance and peer review

Not commissioned, externally peer-reviewed.
